# *Sphaerochara canadensis* (Charophyceae): A circumpolar species with a high temperature optimum

**DOI:** 10.1111/jpy.70111

**Published:** 2025-12-03

**Authors:** Julien Böhm, Irmgard Blindow, Niclas Gyllenstrand, Wolfgang Diewald, Hendrik Schubert

**Affiliations:** ^1^ Department of Aquatic Ecology, Institute of Biosciences University of Rostock Rostock Germany; ^2^ Biological Station of Hiddensee, Experimental Plant Ecology University of Greifswald Greifswald Germany; ^3^ Swedish Museum of Natural History Stockholm Sweden; ^4^ Büro für Botanik Straubing Germany

**Keywords:** Charophyceae, climate change, eDNA, light, PAM, photosynthesis, physiology, Sphaerochara, temperature

## Abstract

*Sphaerochara canadensis*, an aquatic macrophyte belonging to the Characeae, is described as a species with a circumpolar distribution and occurs in the polar (to boreal) zonobiomes, suggesting that it is cold‐stenothermic. A recent report of an occurrence in Lake Wolfgangsee, Austria, contradicted this assumption, prompting this study to investigate the species' physiological adaptation capabilities and, consequently, ability to survive in non‐polar environments. Field measurements at Lake Torneträsk, Sweden, indicated that *S. canadensis* is more adapted to low light compared to the co‐occurring charophytes, with water temperatures in the lake ranging from 10 to 12°C during the experiment. Cultivation experiments also revealed clear temperature effects on growth, photosynthetic performance, and pigment composition at 5, 10, 15, and 20°C, with higher temperatures having a positive impact. Furthermore, it was shown that the species can adapt to different light intensities. A published occurrence of the species in Austria is probably erroneous. The photographic material of the original report was misidentified, and eDNA analyses of water samples taken from Lake Wolfgangsee and surrounding water bodies failed to confirm the presence of the species. Consequently, a factor other than temperature limits its distribution. Identifying the factor is crucial for conservation under climate change.

**Abbreviations**
alpha: initial slope of the light curve
ANOVA: analysis of variance
car: carotenoids
Chl *a*
: chlorophyll a
Chl *b*
: chlorophyll *b*
eDNA: environmental DNA
ETR_max_
: maximum electron transport rate
HL: high light
*I*_HS NPQ_
: irradiance at which half‐saturation of NPQ_max_ is reached
I_k_
: transition point from light limitation to light saturation
LL: low light
ML: mid light
NPQ: non‐photochemical quenching
NPQ_max_
: maximum non‐photochemical quenching
PAM: pulse‐amplitude‐modulated
PCR: polymerase chain reaction
PI: photosynthesis‐irradiance
PS I: photosystem I
PS II: photosystem II
Y(I): yield of Photosystem I
Y(II): yield of Photosystem II

## INTRODUCTION

Characeae have a global distribution (Wood & Imahori, 1965). However, the extent of the distribution range varies greatly among species. Although some, like *Chara globularis*, are cosmopolitan with widespread distribution (Zviedre & Schubert, 2024), others, such as *Sphaerochara canadensis*, have much smaller known ranges or limited recorded occurrences (Langangen, 2024). *Sphaerochara canadensis* is the only species of Characeae known to have a restricted circumpolar distribution (Langangen, 2024). The first record of the species from Lake Superior, Ontario, Canada, was described as *Tolypella canadensis* (Sawa, 1973). Recently, the name *Sawia canadensis* (Romanov, 2025) has been proposed, but the morphologic characters put forward to establish *Sawia* are, in our opinion, not sufficiently distinct from those of *Sphaerochara*. With increasing interest in charophytes, several occurrences from Alaska, Finland, Greenland, Iceland, Norway, Russia, and Sweden have been confirmed (Hrafnsdottir et al., 2019; Langangen, 1993, 2001; Langangen et al., 1996; Langangen & Blindow, 1995; Langangen & Zhakova, 2002; Romanov & Kopyrina, 2016). According to the map in Langangen (2024), most sites have been located in the polar zonobiome, with some in the boreal, characterized by low summer temperatures and long winters (Walter & Breckle, 1999). *Sphaerochara canadensis* occurs there in habitats that have relatively low water temperatures (Langangen, 2024). Its occurrence in Lake Superior represents the southernmost known record (Langangen, 2024), at the boundary of the boreal zonobiome (Walter & Breckle, 1999), with water temperatures of 12–17°C during the growing season (Sawa, 1973). Based on its distribution, it was concluded that *S. canadensis* is probably cold‐stenothermic (Langangen, 2024). In 2016, the species was reported from Lake Wolfgangsee, Austria, that is, far outside the known distribution area (Pall et al., 2016). Temperature measurements for the actual site in Lake Wolfgangsee between 1999 and 2016 were up to 21.7°C at depths of 0–12 m (Schaber, 2019). At the reported depth of ~2–4 m by Pall et al. (2016), temperatures are inconsistent with a cold‐stenothermic species and exceed those recorded at the previously southernmost known site, making it unlikely that the species is a glacial relic, despite such relics being common among alpine plants (Birks, 2008).

The following hypotheses have been proposed: (1) *Sphaerochara canadensis* is a physiologically cold‐stenothermic species, and (2) *S. canadensis* is restricted to cold (polar to boreal) zonobiomes in Europe. We investigated the growth and physiological response of *S. canadensis* to different temperatures and light conditions to gain a deeper understanding of the reaction to altered environmental conditions and assess the condition of the plant, both at Lake Torneträsk, northern Sweden, and in the laboratory. Furthermore, the possible occurrence in Austria was checked by an eDNA study.

## MATERIALS AND METHODS

### Sampling site

Material for field measurements and the laboratory experiment was collected at Lake Torneträsk, Sweden (Figure S1). The lake is 70 km long and up to 10 km wide, with a surface area of 330 km^2^. The average depth is 53 m, with a maximum depth of 168 m (Meyer‐Jacob et al., 2017). From January to May, the lake is covered by ice (Callaghan et al., 2010). The lake is classified as oligotrophic (Sveriges lantbruksuniversitet, 2021).

### Collecting of material

Stands of Characeae were located with an underwater drone (M2 PRO, Chasing, Guangdong, China) at three different sites (site 1: 68.35699, 18.82280; site 2: 68.3558605, 18.8408722; and site 3: 68.3619216, 18.8431646) close to the Abisko Scientific Research Station. All individuals for the experiments were taken from site 2. The depth and temperature of the sites were recorded by the drone. Sampling by means of an Ekman–Birge soil grab and a rake took place in August 2023. Individuals were identified by use of a binocular at the field station.

### Field measurement of photosynthetic performance

Samples of *Sphaerochara canadensis* and *Nitella opaca* were immediately dark‐adapted after sampling at the same water depth during the evening hours and transported to the local laboratory. Photosynthetic performance of the individuals was measured after 12 h of pre‐acclimation in darkness, simulating the night period. A photosynthesis–irradiance (PI) curve for the photosystem II (PS II) was then recorded using a pulse‐amplitude modulation (PAM) chlorophyll fluorometer (JUNIOR‐PAM, Heinz Walz, Effeltrich, Bavaria, Germany). The PI curve consisted of 11 irradiation steps (0, 25, 45, 65, 90, 125, 190, 285, 420, 625, and 820 μmol photons · m^−2^ · s^−1^), with each irradiance step lasting 60 s. Maximum electron transport rate (ETR_max_), initial slope (alpha), and transition point from light limitation to light saturation (*I*_k_) were then calculated by fitting the PI curve to the model of Eilers and Peeters (1988) using the least squares method by the Solver add‐in in Excel (Version 16.0, Microsoft, 2021). The effect of photoinhibition was incorporated into all calculations.

### Field measurement of attenuation

Underwater scalar irradiance was measured using a high‐resolution spectroradiometer (SR‐9910; Macam Photometrics, Glasgow, Scotland, United Kingdom) fitted with a 0.7‐cm diameter spherical light collector via a 10‐m long optical fiber. To calculate the diffuse attenuation coefficient *K*_o_ (*λ*; Smith, 1968), successive underwater scans were carried out at depth intervals of 0.5 m between 0.5 and 4 m.

### Transport of material

Plant material was transported in temperature‐controlled containers (CFF 70DZ, Dometic, Solna, Stockholms län, Sweden) at 10°C to the University of Rostock, Germany, for the cultivation experiment. Several specimens of *Sphaerochara canadensis* and *Nitella opaca* were prepared and have been stored in the herbarium of the University of Rostock (ROST). Images of *S. canadensis* are shown in Figure S2. All relevant locations are shown in Figure S1.

### Cultivation

**FIGURE 1:**
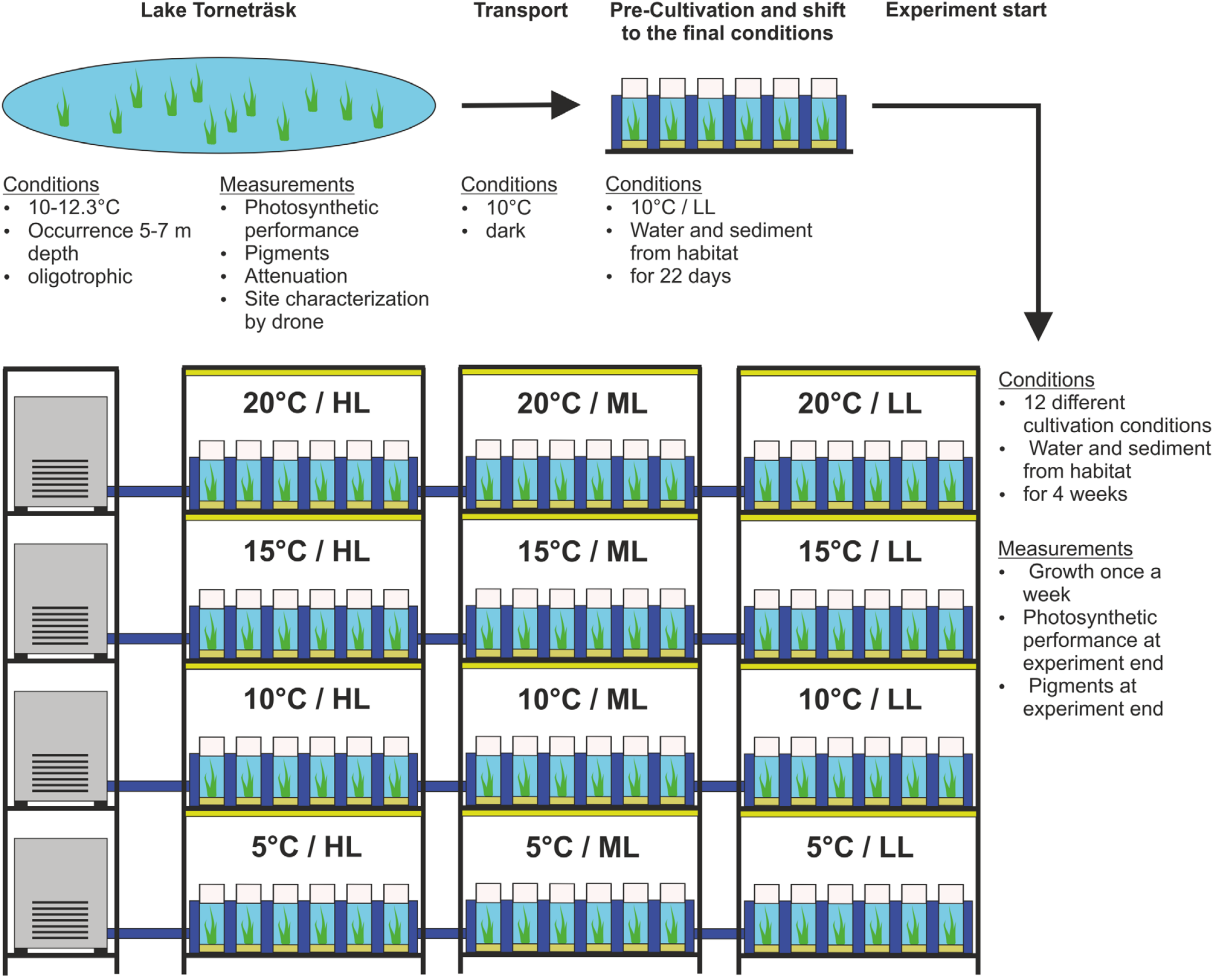
Experimental timeline and cultivation system setup.

The cultivation system had four temperature levels (5, 10, 15, and 20°C) as well as three light levels, which have been referred to as high light (HL), mid light (ML), and low light (LL). This resulted in 12 different cultivation conditions. Each individual was planted in a cultivation vessel (Zylinder‐Glas 600 mL, J. Weck, Wehr‐Öflingen, Baden‐Württemberg, Germany) with habitat sediment and 430 mL of habitat water. All cultures were pre‐cultivated for 22 days under LL at 10°C and then shifted to their final 12 experimental conditions. The cultures were placed in tubs (customized design, Kunststoffverarbeitung Tino Beckmann, Gomaringen, Baden‐Württemberg, Germany) through which cooling water was pumped (DC Runner 2.3, Aqua Medic, Bissendorf, Lower Saxony, Germany). The water was tempered by coolers (Titan 600/1500/2200, Aqua Medic, Bissendorf, Lower Saxony, Germany). The temperature in the culture vessels was measured by loggers (UA‐002‐64, Onset, Bourne, Massachusetts, United States) every 15 min. These resulted in the following actual temperatures: 5°C = 4.83°C; 10°C = 9.97°C; 15°C = 15.38°C; and 20°C = 19.95°C (median values; Figure S3). Light‐emitting diodes (LEDs; model L35; spectrum NS12; Valoya, Helsinki, Uusimaa, Finland) were used to illuminate the cultivation. Dimmed light was generated by turning the LEDs. A light meter (LI‐250A, LI‐COR Biosciences, Lincoln, Nebraska, United States) was used to measure the exact photon flux density at cooling water level at the position of each cultivation vessel. These resulted in HL = 243.5 μmol photon · m^−2^ · s^−1^; ML = 130 μmol photons · m^−2^ · s^−1^, and LL = 102 μmol photons · m^−2^ · s^−1^ (median values; Figure S3). The spectrum of each light level was also determined using a spectroradiometer (SR‐9910; Macam Photometrics, Glasgow, Scotland, United Kingdom; Figure S3). The day:night rhythm was set to 12:12 h. The experimental setup is shown in Figure 1.

### Growth analysis

Growth was documented on a weekly basis with a camera fixed at a specific distance from the cultivation vessel. A scale was placed in front of and behind the glass. The captured image was then measured with ImageJ (Schneider et al., 2012). To do this, the scale was calibrated to the center of the vessel using the front and rear scales. The longest part of the alga was measured using the Segmented Line function. Fresh weight was determined at the end of the experiment using a scale (Entris II BCE641‐1S, Sartorius AG, Göttingen, Lower Saxony, Germany).

### Lab measurement of photosynthetic performance

The samples were dark‐acclimated in their cultivation vessels for at least 30 min. Temperature‐controlled containers were used to maintain the cultivation temperature during acclimatization. A Dual PAM chlorophyll fluorometer/absorption spectrometer (DUAL‐PAM‐100, Heinz Walz GmbH, Bavaria, Effeltrich, Germany) was then used to analyze the photosynthetic performance. This facilitates the parallel detection of PS II with a PAM chlorophyll fluorometer and photosystem I (PS I) with an absorption spectrometer with two wavelengths. For the measurement, whole algae were placed in a quartz cuvette with habitat water. Care was taken to ensure that the apical part was in the measurement field. To keep the sample at cultivation temperature during the measurement, the Dual‐PAM was equipped with a water cooler system (WKL 26, Thermo Fisher Scientific, Waltham, Massachusetts, United States), and the temperature was observed by a thermometer (Inside/outside thermometer ROTILABO®, Carl Roth, Karlsruhe, Baden‐Württemberg, Germany). A PI curve with 11 irradiation steps (0, 40, 49, 62, 112, 183, 237, 391, 603, 928, and 1420 μmol photons · m^−2^ · s^−1^) was then recorded. The time set for each irradiance was 60 s up to 62 μmol photons · m^−2^ · s^−1^ and 30 s thereafter. The parameters ETR_max_ and *I*_k_ were then calculated by fitting the PI curve to the model of Eilers and Peeters (1988) by using the package pam (Böhm & Schrag, 2025) in R (R Core Team, 2024). The effect of photoinhibition was incorporated into all calculations. The package minpack.lm (Elzhov et al., 2023) was used for all subsequent regressions. Maximum non‐photochemical quenching (NPQ_max_) and the irradiance at whcih half‐saturation of NPQ_max_ is reached (*I*_HS NPQ_) were modeled using the Michaelis–Menten equation (Michaelis & Menten, 1913). To calculate the initial Y(II)/Y(I) ratio, a linear regression was performed by using irradiance steps from 40 to 391 μmol photons · m^−2^ · s^−1^. The quality of the regression was assessed using *R* ^2^ and regressions with *R* ^2^ < 0.7 were excluded.

### Pigment analysis

The pigment analysis of the laboratory cultures was carried out directly after the photosynthesis measurements. Plants collected in the field were frozen at −20°C and then transported to the laboratory at the University of Rostock. The apical part was used for the analysis. Chlorophyll *a* (Chl *a*), chlorophyll *b* (Chl *b*), and carotenoids (car) were extracted with dimethylformamide (DMF) for 24 h, at −20°C in the dark. Subsequently, the entire supernatant without tissue residues was transferred to a 1 cm quartz cuvette, and the absorbance from 350 nm to 750 nm was recorded using a spectrophotometer (UV/VIS spectrometer Lambda 2, PerkinElmer, Waltham, Massachusetts, United States). The pigment concentrations were then determined according to Wellburn (1994).

### eDNA sampling and analysis

eDNA water samples were taken from 12 different locations in November 2023. The sampling design was as follows (Table S1): Sample 4 was taken at the presumed location of *Sphaerochara canadensis* in Lake Wolfgangsee, sample 3 at other locations within the same lake, and sample 5 at the outlet of Lake Wolfgangsee. Sample 1 was taken from Lake Traunsee. All water from Lake Wolfgangsee, Lake Hallstätter See, Lake Altaussee, Lake Grundlsee, and Lake Toplitzsee flows into this lake. Sample 2 was collected from Lake Attersee, which is part of a separate water system together with Lake Mondsee. The water temperatures at the sampling sites were between 9 and 11°C.

DNA was extracted from Sylphium‐filters by adding 3 mL ATL buffer (Qiagen, Venlo, Limburg, Netherlands) and 25 μL Proteinase K followed by vortexing and incubation ON at 56°C. After incubation, the lysate was extracted using the MagBind® Plant DNA HDQ 96 kit (Omega Bio‐tek, Norcross, Georgia, United States) and a Kingfisher Duo extraction robot (Thermo Fisher Scientific, Waltham, Massachusetts, United States) using the manufacturer's instructions. Following extractions, the samples were tested for polymerase chain reaction (PCR) inhibition by spiking with exogenous DNA. For detection of *Sphaerochara canadensis* the samples were run on a real‐time PCR detection system (CFX96, Bio‐Rad Laboratories, Hercules, California, United States) using the following PCR profile: Initial denaturation at 95°C for 10 min followed by 50 cycles of 95°C for 15 s and then 60°C for 60 s. The PCR protocol used included 6.25 μL TaqMan™ Environmental master mix 2.0 (Thermo Fisher Scientific, Waltham, Massachusetts, United States), 0.5 μL of each primer and probe listed in Table 1, 0.75 μL H_2_0, and 4 μL of DNA extract. Results were analyzed using the CFX Maestro software (Bio‐Rad Laboratories, Hercules, California, United States). All samples were run in triplicates, and positive and negative controls were included.

**TABLE 1:** Sequences of primers and samples for eDNA analysis.

Primers/probes	Sequence 5′➔3′
S_canadensis_FWD	CTTTCGCGCCGTCTTCCG
S_canadensis_REV	TCAAGAGGACGTGTGGGAC
S_canadensis_probe	[6FAM]‐GGCCCTCCCGTCCCAGTTCT‐[BHQ1]

### Statistical evaluation and data visualization

The statistical analysis and graphical representation of the data were conducted using R (R Core Team, 2024). At least five individuals of each species were used in the field experiment. In the cultivation experiment, six individuals per group were used. In some cases, the number of measurement points per group was lower. The R packages readxl (Wickham & Bryan, 2015), data.table (Barrett et al., 2006), ggthemes (Arnold, 2012), ggplot2 (Wickham, 2016), ggsignif (Ahlmann‐Eltze & Patil, 2017), patchwork (Pedersen, 2024), and tidyverse (Wickham et al., 2019) were utilized for data processing and visualization. An approximative general independence test was used to assess differences between two groups (field experiment) using coin (Hothorn et al., 2006). A two‐factorial permutation analysis of variance (ANOVA) was used to assess the effects of light, temperature, and their interaction (cultivation experiment) using permuco (Frossard & Renaud, 2021). The tests were performed with 1,000,000 permutations to estimate the *p*‐value. Results were considered significant at *p* < 0.05.

## RESULTS

**FIGURE 2:**
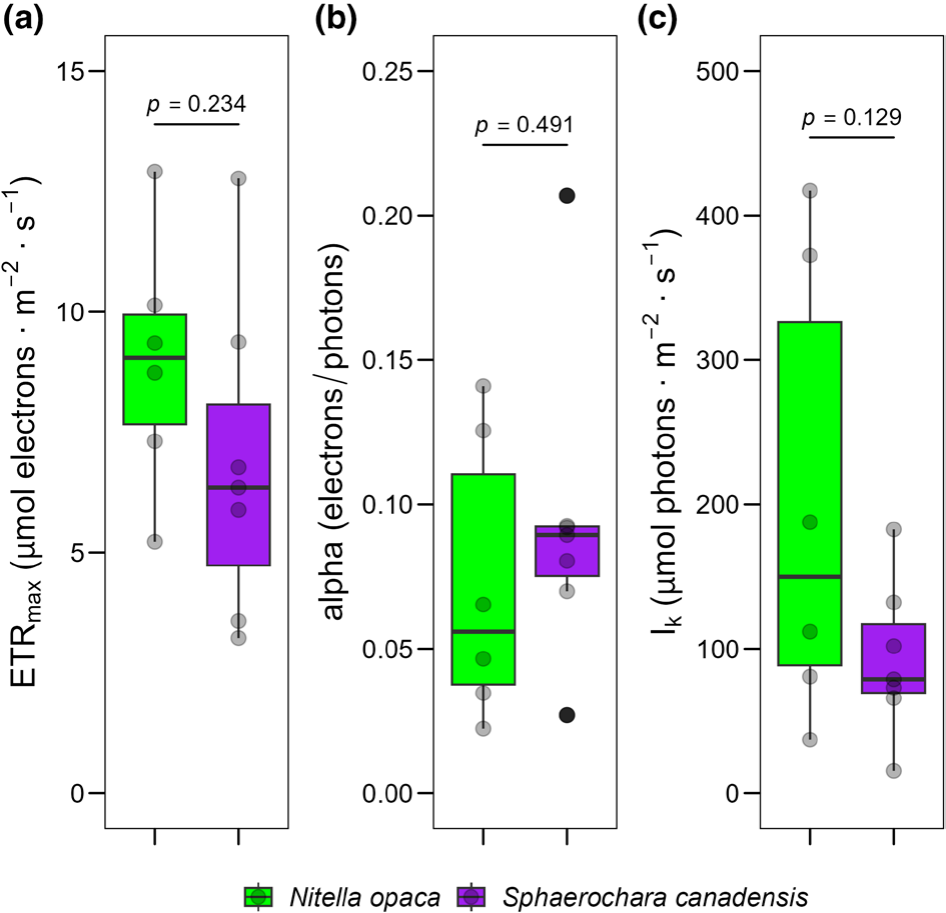
Photosynthetic performance of PS II of *Nitella opaca* and *Sphaerochara canadensis* (*S. canadensis*) at Lake Torneträsk. (a) ETR_max_ (μmol electrons · m^−2^ · s^−1^); (b) Alpha (electrons/photons); (c) *I*_k_ (μmol photons · m^−2^ · s^−1^). The box plots cover the 25th–75th percentiles, with black lines indicating the median. Whiskers extend to 1.5 times the interquartile range, with outliers shown as black dots. Semi‐transparent black dots represent individual data points. *p*‐values above the boxplots indicate statistical differences between groups (Approximate General Independence Test, *p* < 0.05 considered significant).

**FIGURE 3:**
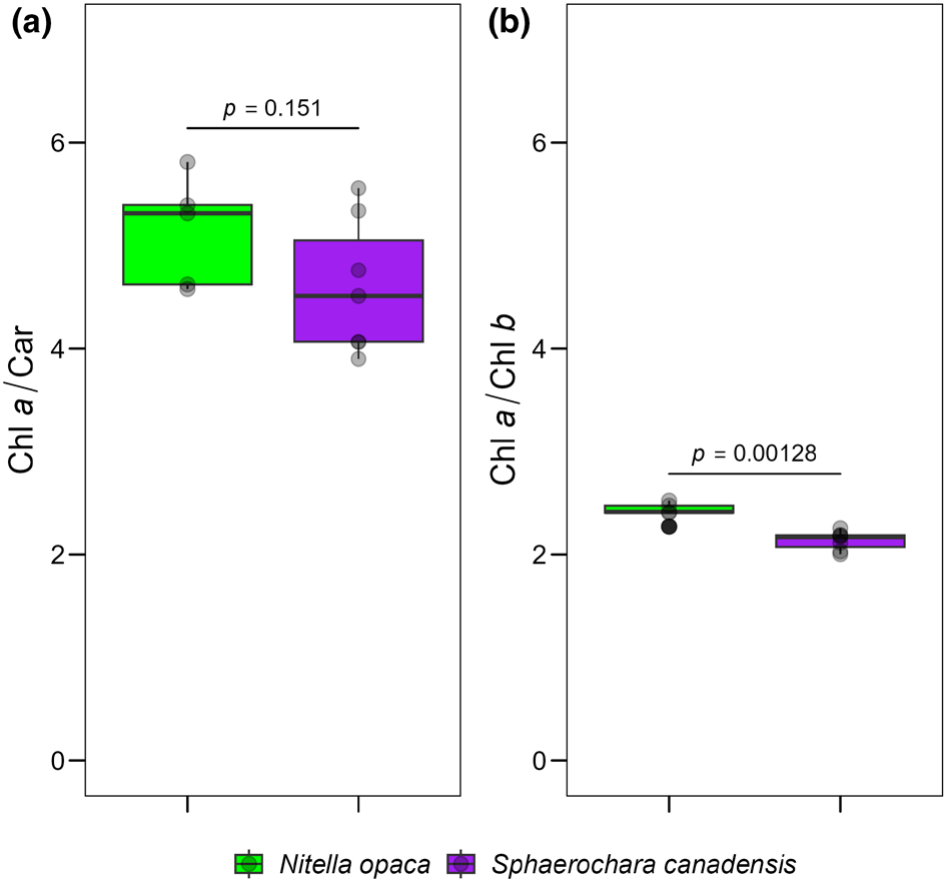
Relative pigment ratios of *Nitella opaca* and *Sphaerochara* canadensis at Lake Torneträsk. (a) Ratio of Chl *a* to car; (b) Ratio of Chl *a* to chl *b*. The box plots cover the 25th–75th percentiles, with black lines indicating the median. Whiskers extend to 1.5 times the interquartile range, with outliers shown as black dots. Semi‐transparent black dots represent individual data points. *p*‐values above the boxplots indicate statistical differences between groups (Approximate General Independence Test, *p* < 0.05 considered significant).

On‐site measurements of photosynthetic performance showed no significant differences between *Nitella opaca* and *Sphaerochara canadensis* (Approximative General Independence Test; ETR_max_: *p* = 0.23, alpha: *p* = 0.49, *I*_k_: *p* = 0.13; Figure 2). Furthermore, pigment data showed a significantly higher Chl *a* to Chl *b* ratio in *N. opaca* (Approximative General Independence Test: *p* = 0.0013; Figure 3).

**FIGURE 4:**
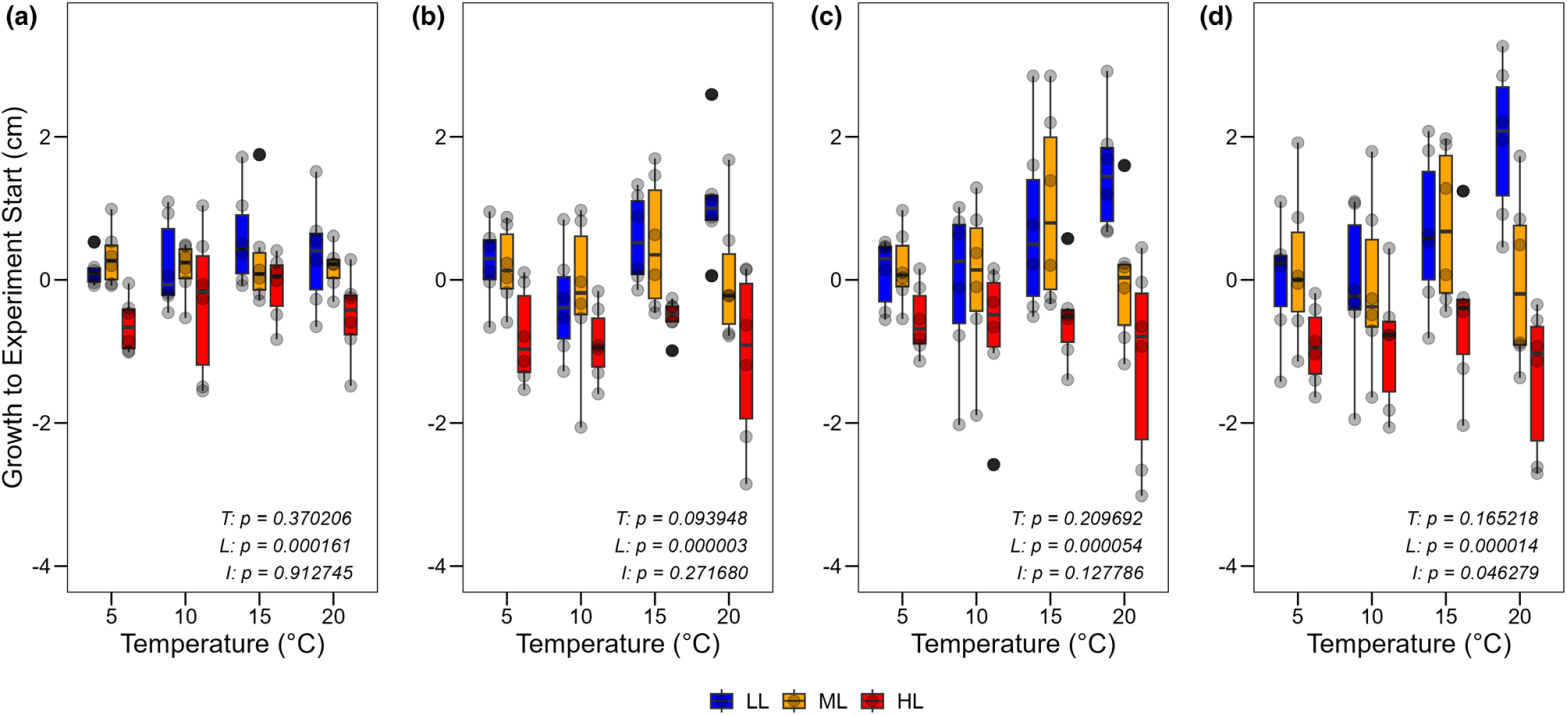
Absolute length growth compared to the start of the experiment of *Sphaerochara canadensis* in culture under different temperature and light conditions. (a) After 1 week; (b) After 2 weeks; (c) After 3 weeks, (d) After 4 weeks. The box plots cover the 25th–75th percentiles, with black lines indicating the median. Whiskers extend to 1.5 times the interquartile range, with outliers shown as black dots. Semi‐transparent black dots represent individual data points. Statistical results for the main effects of temperature (*T*), light (*L*), and interaction effect (*I*) at each timepoint are shown separately at the bottom right (permutation ANOVA, *p* < 0.05 considered significant).

All individuals survived the cultivation experiment. Over a period of 4 weeks, the cultures under HL had reduced their size, while cultures under ML showed hardly any change in length. Individuals under LL showed length growth, which was more distinct with higher temperature, as indicated by median values, with a significant effect of light (permutation ANOVA; week 1: *F*_2,60_ = 9.89, *p* = 0.00016; week 2: *F*_2,60_ = 15.78, *p* = 0.000003; week 3: *F*_2,60_ = 11.55, *p* = 0.000054; week 4: *F*_2,60_ = 14.19, *p* = 0.000014) observed in all weeks and a significant interaction between light and temperature appearing after 4 weeks (permutation ANOVA; *F*_6,60_ = 2.30, *p* = 0.046; Figure 4). None of the individuals developed gametangia, including three additional cultures maintained at 10°C and LL for 12 months.

**FIGURE 5:**
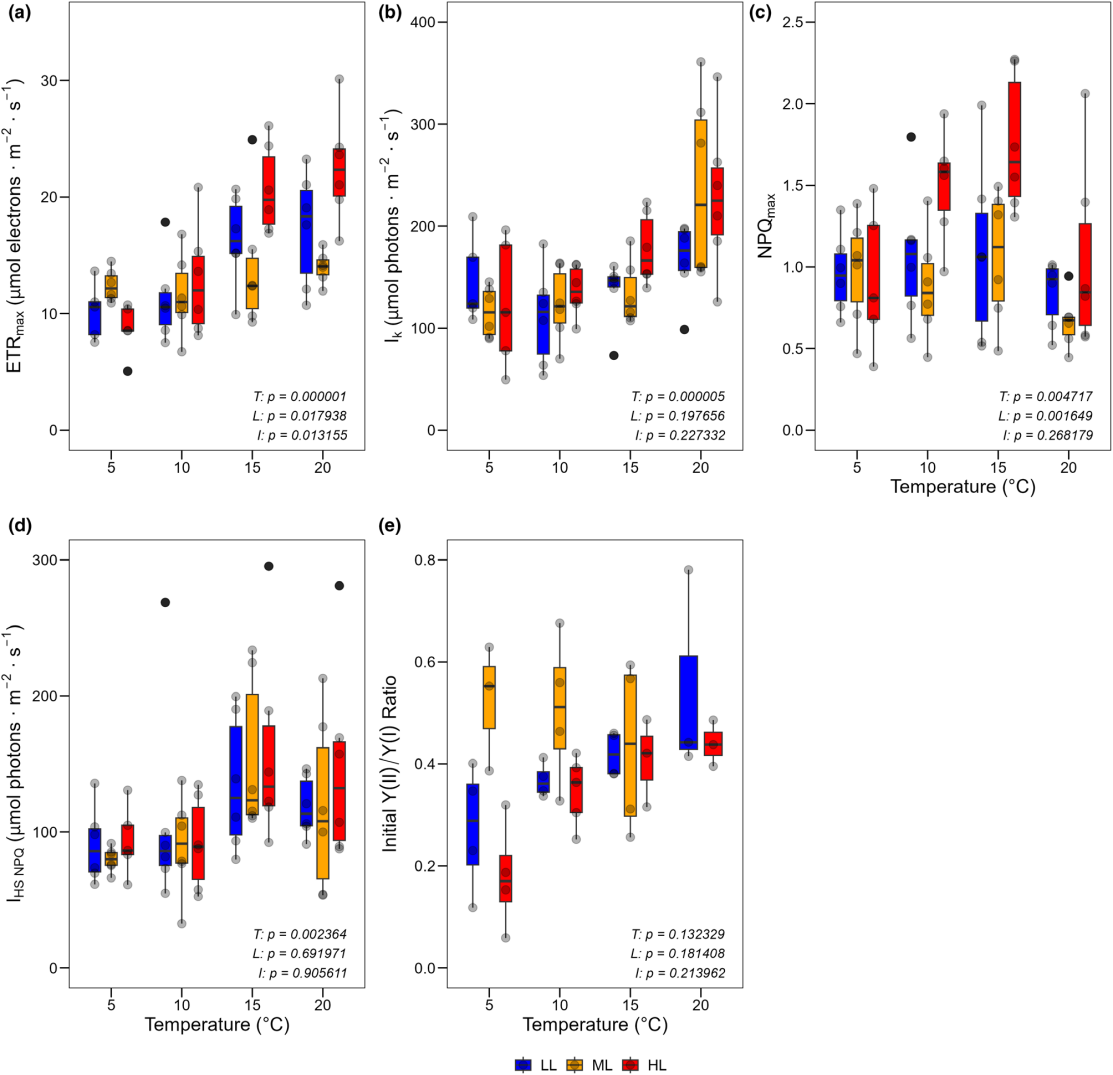
Photosynthesis performance of *Sphaerochara canadensis* after four weeks of cultivation under different temperature and light conditions. (a) ETR_max_ (μmol electrons · m^−2^ · s^−1^); (b) *I*_k_ (μmol photons · m^−2^ · s^−1^); (c) NPQ_max_; (d) *I*_HS NPQ_ (μmol photons · m^−2^ · s^−1^); (e) Initial Y(II)/Y(I) Ratio. The box plots cover the 25th–75th percentiles, with black lines indicating the median. Whiskers extend to 1.5 times the interquartile range, with outliers shown as black dots. Semi‐transparent black dots represent individual data points. Statistical results for the main effects of temperature (*T*), light (*L*), and interaction effect (*I*) are shown at the bottom right (permutation ANOVA, *p* < 0.05 considered significant).

Photosynthetic performance measurements at the end of the cultivation experiment revealed that ETR_max_ was significantly influenced by temperature, light, and their interaction (permutation ANOVA; temperature: *F*_3,58_ = 16.14, *p* = 0.000001; light: *F*_2,58_ = 4.31, *p* = 0.018; interaction: *F*_6,58_ = 2.99, *p* = 0.013). With higher temperature, the median values rose, with cultures under ML showing a weaker response to temperature changes. Median ETR_max_ values at 20°C and HL were twice as high as those at 5°C, reaching the overall highest median values (Figure 5). A similar pattern was observed for *I*_k_, with cultures under ML responding similarly to those under HL and LL, but only the effect of temperature was significant (permutation ANOVA; *F*_3,58_ = 11.82, *p* = 0.000005). As with ETR_max_, the lowest median values were recorded at lower temperatures, whereas the highest median values occurred at 20°C. Under this condition, the highest median *I*_k_ values were in plants under ML and HL (Figure 5). For NPQ_max_, both temperature and light had significant effects (permutation ANOVA; temperature: *F*_3,59_ = 4.82, *p* = 0.0047; light: *F*_2,59_ = 7.07, *p* = 0.0016). Under HL, median NPQ_max_ values increased from 5 to 15°C but then decreased back to the 5°C level at 20°C (Figure 5). For *I*_HS NPQ_, a significant temperature effect was observed (permutation ANOVA; *F*_3,59_ = 5.38, *p* = 0.0024), with median values increasing at higher temperatures (Figure 5). Finally, no significant influence on the initial Y(II)/Y(I) ratio was observed (permutation ANOVA; temperature: *F*_3,30_ = 1.45, *p* = 0.13; light: *F*_2,30_ = 0.93, *p* = 0.18; interaction: *F*_6,30_ = 1.27, *p* = 0.21). However, median values of the ratio tended to increase with higher temperatures under HL and LL. Under HL, the median ratio doubled between 5°C and 20°C (Figure 5).

**FIGURE 6:**
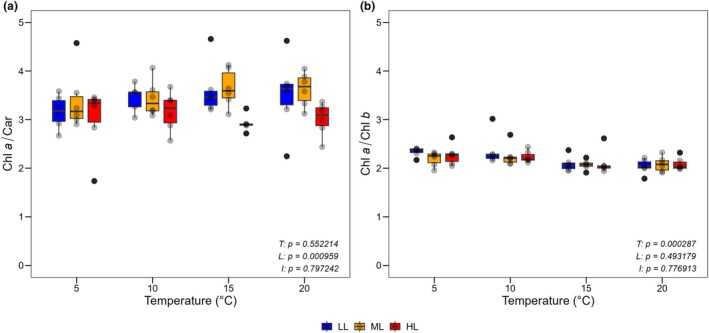
Relative pigment ratios of *Sphaerochara canadensis* in culture under different temperature and light conditions. (a) Ratio of chl *a* to car; (b) Ratio of Chl *a* to Chl *b*. The box plots cover the 25th–75th percentiles, with black lines indicating the median. Whiskers extend to 1.5 times the interquartile range, with outliers shown as black dots. Semi‐transparent black dots represent individual data points. Statistical results for the main effects of temperature (*T*), light (*L*), and interaction effect (*I*) are shown at the bottom right (permutation ANOVA, *p* < 0.05 considered significant).

Analyzed pigment data showed a significant effect of light on the Chl *a* to car ratio (permutation ANOVA; *F*_2,59_ = 7.43, *p* = 0.00096; Figure 6). A significant temperature effect was observed for the Chl *a* to Chl *b* ratio (permutation ANOVA; *F*_3,59_ = 7.13, *p* = 0.00029), with two clusters identified across all light levels: one at 5 and 10°C and the other at 15 and 20°C. Median values of the ratio were slightly lower at 15 and 20°C (Figure 6).

Image footage from Lake Wolfgangsee (Pall et al., 2016) showed divided sterile branchlets, which ruled out *Sphaerochara canadensis*. The individual is probably *Nitella mucronata*, according to the determination key presented by van de Weyer et al. (2024). Furthermore, in none of the water samples taken from Lake Wolfgangsee and the surrounding lakes, did eDNA‐analyses identify the presence of *S. canadensis* (Table S1).

## DISCUSSION

*Sphaerochara canadensis* was observed together with *Nitella opaca* in the clear‐water lake Lake Torneträsk at a depth of 5–7 m at a temperature of 10–12.3°C (Figures S4 and S5). Consequently, the lake represents a typical habitat of *S. canadensis* (Langangen & Zhakova, 2002). Pigment data from individuals taken from the same water depth in the lake suggest that *S. canadensis* is adapted to lower light conditions compared to *N. opaca*, as indicated by the significantly lower Chl *a* to Chl *b* ratio in *S. canadensis*. The Chl *a* to Chl *b* ratio reflects the adaptation to habitat light intensity by adjusting the PS I/PS II ratio and the size and composition of the light‐harvesting complexes (LHCs) of the photosystems, and it decreases under low light intensity (Porra, 2002). Our results indicate that Characeae have species‐specific Chl *a* to Chl *b* ratios independent of light conditions. For three other charophyte species, Küster et al. (2004) have already shown that this ratio is light‐adapted only to a limited extent or not at all.

In the laboratory experiment, cultures under HL reduced their size, whereas plants under LL showed increased length growth but only at higher temperatures. This could be explained by shoot elongation caused by light deficiency, as observed in *Chara aspera*. Here, shoot elongation was observed at low irradiance, whereas biomass increase was favored by higher irradiances (Blindow et al., 2003). For *Sphaerochara canadensis*, the weight‐to‐length ratio decreased with lower light intensities (Figure S6), indicating that the higher length increase at lower light intensities was caused by shoot elongation rather than biomass increase. The interaction with temperature suggests that this shoot elongation is more efficient at higher temperatures and indicates a greater capacity to adjust to low light intensity under warmer conditions, as shoot elongation represents an adaptation to low light availability in turbid lakes (Blindow & Schütte, 2007).

With higher temperature, the photosynthesis parameters ETR_max_ and *I*_k_ were both higher, indicating that the available light energy can be utilized more fully at higher temperatures. A clear temperature effect on NPQ_max_, the maximum non‐photochemical quenching, was observed, but only under HL, with NPQ_max_ increasing from 5 to 15°C. This was expected, as NPQ is a protective mechanism against damage caused by excessive light energy (Bassi & Dall'Osto, 2021). Changes in the Chl *a*/car ratio further indicated the ability to adjust for photoprotection, as has been shown for other Characeae species (Küster et al., 2004). A temperature‐dependent effect was also detected for *I*_HS NPQ_, the light intensity at which the half‐saturation of NPQ is achieved. This effect suggests that with increasing temperatures, NPQ only becomes relevant at higher irradiance, allowing light energy to be more effectively utilized for photochemical quenching at relatively higher light intensities. This idea was supported by the previously discussed *I*_k_ data. Furthermore, this result suggests that *Sphaerochara canadensis* can effectively protect itself against stronger irradiance, aligning with its documented occurrence in shallow waters and down to depths of 17 m (Langangen, 2024). The Chl *a* to Chl *b* ratio changed with temperature but not with light, confirming limited light‐driven adjustments in Characeae (Küster et al., 2004).

Consequently, the photosynthesis‐related data show that with increasing temperature, the available light energy can be utilized more efficiently by *Sphaerochara canadensis*. This response is similar to those of other aquatic plants, which reach optimal photosynthesis rates at relatively high water temperatures (20–35°C; Bornette & Puijalon, 2011). Water temperature affects productivity by influencing the rate at which chemical reactions proceed, usually at an optimum rate (Carr et al., 1997). However, it remains speculative which specific photosynthetic processes are limited by reduced temperature, as it has been shown that land plants respond differently to temperature changes depending on their photosynthetic pathways (C3, C4, CAM; Yamori et al., 2014). The functionality of a possible carbon‐concentration mechanism (CCM) in Characeae is not yet fully understood and is currently being studied in *Chara braunii* (Heise et al., 2024).

At temperatures as high as 20°C, higher length growth as well as a more efficient photosynthetic exploitation of the available light energy was observed. We therefore rejected our first hypothesis that *Sphaerochara canadensis* is physiologically cold‐stenothermic. Studies with *Fontinalis antipyretica* and *Callitriche hamulata* have already shown that macrophytes collected from polar regions have temperature optima for growth and photosynthetic performance far above the conditions of their sampling sites, with the key difference being that these species have a broader distribution than *S. canadensis* (Lauridsen et al., 2020; Maberly, 1985).

We could not reject our second hypothesis that *Sphaerochara canadensis* is restricted to cold (polar to boreal) zonobiomes in Europe, as the only report from outside these regions appears to be a misidentification. Furthermore, during a diving survey in Lake Wolfgangsee, the species could not be located (Hohla & Diewald, 2024). As a result of our investigation, this assessment was incorporated into the project “Charophytes of Austria,” in which an occurrence in Lake Wolfgangsee was similarly deemed unlikely (Hohla et al., 2025).

The physiological data did not provide an explanation for the restricted distribution of the species. One possible explanation for this restriction is that gametangia are only formed under extreme photoperiods, such as those in polar regions, which was supported by the fact that no gametangia were observed during a day:night rhythm of 12:12 h. Light and temperature have been shown to influence sexual reproduction in charophytes. *Chara hispida* exhibited an earlier onset and longer reproductive season in shallower water, where light exposure is higher, compared to deeper waters (Calero et al., 2017). This result is consistent with findings in *C. australis*, for which the transfer of individuals from deeper to shallower water increased the number of reproductive specimens (Casanova, 1994), and experiments with *C. vulgaris*, which demonstrated that higher irradiance and longer day lengths led to an earlier onset of reproduction (Wang et al., 2008). In contrast, *C. braunii* formed the highest number of gametangia under low‐light conditions at 10 μmol photons · m^−2^ · s^−1^, with fewer gametangia produced at higher light intensities, and none at 70 μmol photons · m^−2^ · s^−1^ (Sato et al., 2014). This demonstrated that the requirements for gametangia development may be species‐specific. Competition from other macrophytes may be another explanation for the restricted distribution of *Sphaerochara canadensis*. Climate change influences the distribution range of many species. For 99 species of alpine herbs, butterflies, and birds, a significant shift toward the poles by an average of 6.1 km per decade has been determined (Parmesan & Yohe, 2003). This shift had led to a particular threat for species with a polar distribution by competition from immigrating species (Yasuhara & Deutsch, 2023). The distribution area of *S. canadensis* should therefore be monitored regularly in the future to identify a decline in distribution at an early stage, as already done as part of the Swedish action plan (Zinko, 2017).

## AUTHOR CONTRIBUTIONS

**Julien Böhm:** Conceptualization (equal); data curation (lead); investigation (lead); methodology (lead); software (lead); validation (lead); visualization (lead); writing – original draft (lead); writing – review and editing (equal). **Irmgard Blindow:** Conceptualization (equal); investigation (equal); methodology (equal); validation (equal); writing – review and editing (equal). **Niclas Gyllenstrand:** Investigation (equal); methodology (equal); writing – review and editing (equal). **Wolfgang Diewald:** Investigation (equal); methodology (equal); writing – review and editing (equal). **Hendrik Schubert:** Conceptualization (equal); investigation (equal); methodology (equal); resources (lead); supervision (lead); validation (equal); writing – review and editing (equal).

## Supporting information


**Figure S1.** Overview of sampling and experimental locations. Sampling was carried out from the Abisko Scientific Research Station at Lake Torneträsk. Characeae occurred at sites 1–3, and cultures for the laboratory experiment at the University of Rostock originated from site 2. (Created with QGIS [QGIS Development Team, 2025]).

**Figure S2.** Individuals of *Sphaerochara canadensis* (*S. canadensis*) used in the experiment prior to transplantation into the cultivation vessels.

**Figure S3.** Quantification of the cultivation system. (a) Water temperature (°C) of the cultivation vessels measured every 15 min during the experiment. (b) Photon flux density of PAR (μmol photons · m^−2^ · s^−1^) at the position of the culture vessel measured at cooling water level. (c) Spectrum of the light levels measured in the center and at the edge area of the cooling tubs. The box plots cover the 25th–75th percentiles, with black lines indicating the median. Whiskers extend to 1.5 times the interquartile range, with outliers shown as black dots.

**Figure S4.** Attenuation spectrum of Lake Torneträsk.

**Figure S5.** Temperature and depth profiles recorded during the drone dives. *S. canadensis* was observed between ~5 and 7 m at three different sites, where water temperature ranged from 10°C to 12.3°C. The orange box indicates the area of occurrence.

**Figure S6.** Weight/length ratio (mg · cm^−1^) of *Sphaerochara canadensis* at the end of the cultivation experiment. The box plots cover the 25th–75th percentiles, with black lines indicating the median. Whiskers extend to 1.5 times the interquartile range, with outliers shown as black dots. Semi‐transparent black dots represent individual data points. Statistical results for the main effects of temperature (T), light (L), and interaction effect (I) are shown at the bottom right (permutation ANOVA, *p* < 0.05 considered significant).

**Table S1.** eDNA sampling sites and results.
