# Supplementary figures and images for: Sphaerochara canadensis (Charophyceae): A circumpolar species with a high temperature optimum
Source: J Phycol. 2025 Dec 3;61(6):1863–73. doi: 10.1111/jpy.70111 (PMC12718439; doi:10.1111/jpy.70111)

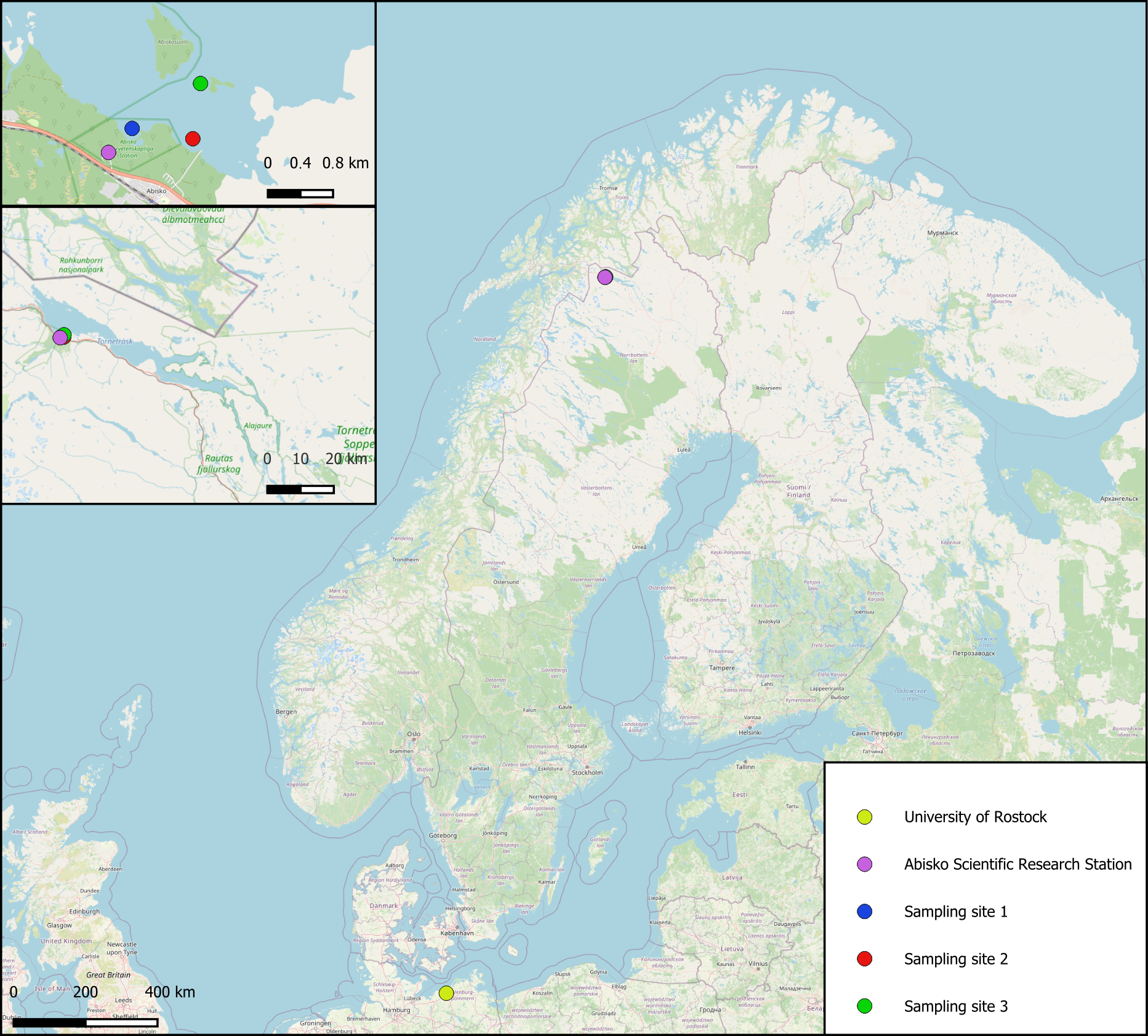

Supplement: Supplementary file 1 — Figure S1. Overview of sampling and experimental locations. Sampling was carried out from the Abisko Scientific Research Station at Lake Torneträsk. Characeae occurred at sites 1–3, and cultures for the laboratory experiment at the University of Rostock originated from site 2. (Created with QGIS [QGIS Development Team, 2025]). [file JPY-61-1863-s007.tif]

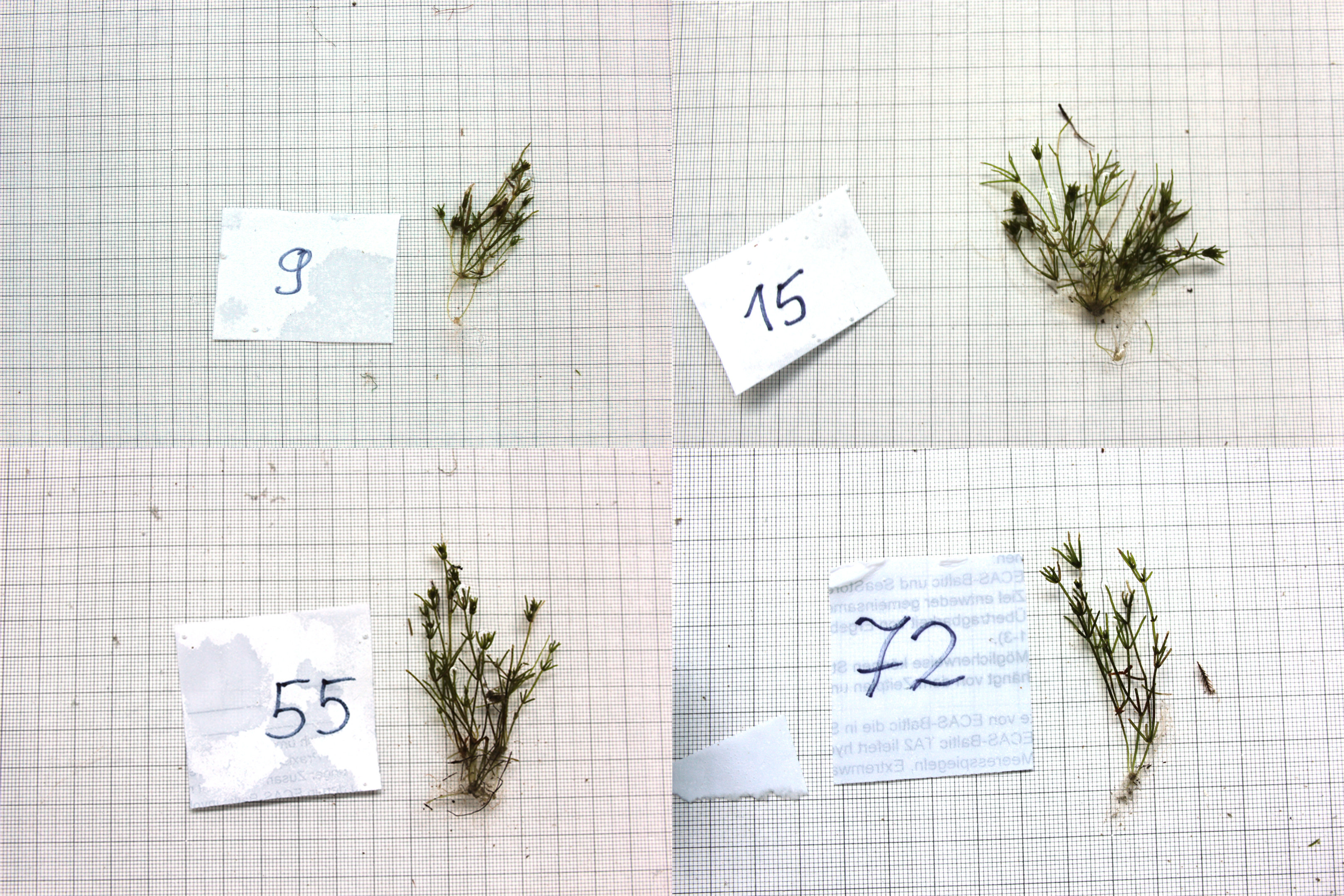

Supplement: Supplementary file 2 — Figure S2. Individuals of Sphaerochara canadensis (S. canadensis) used in the experiment prior to transplantation into the cultivation vessels. [file JPY-61-1863-s004.tif]

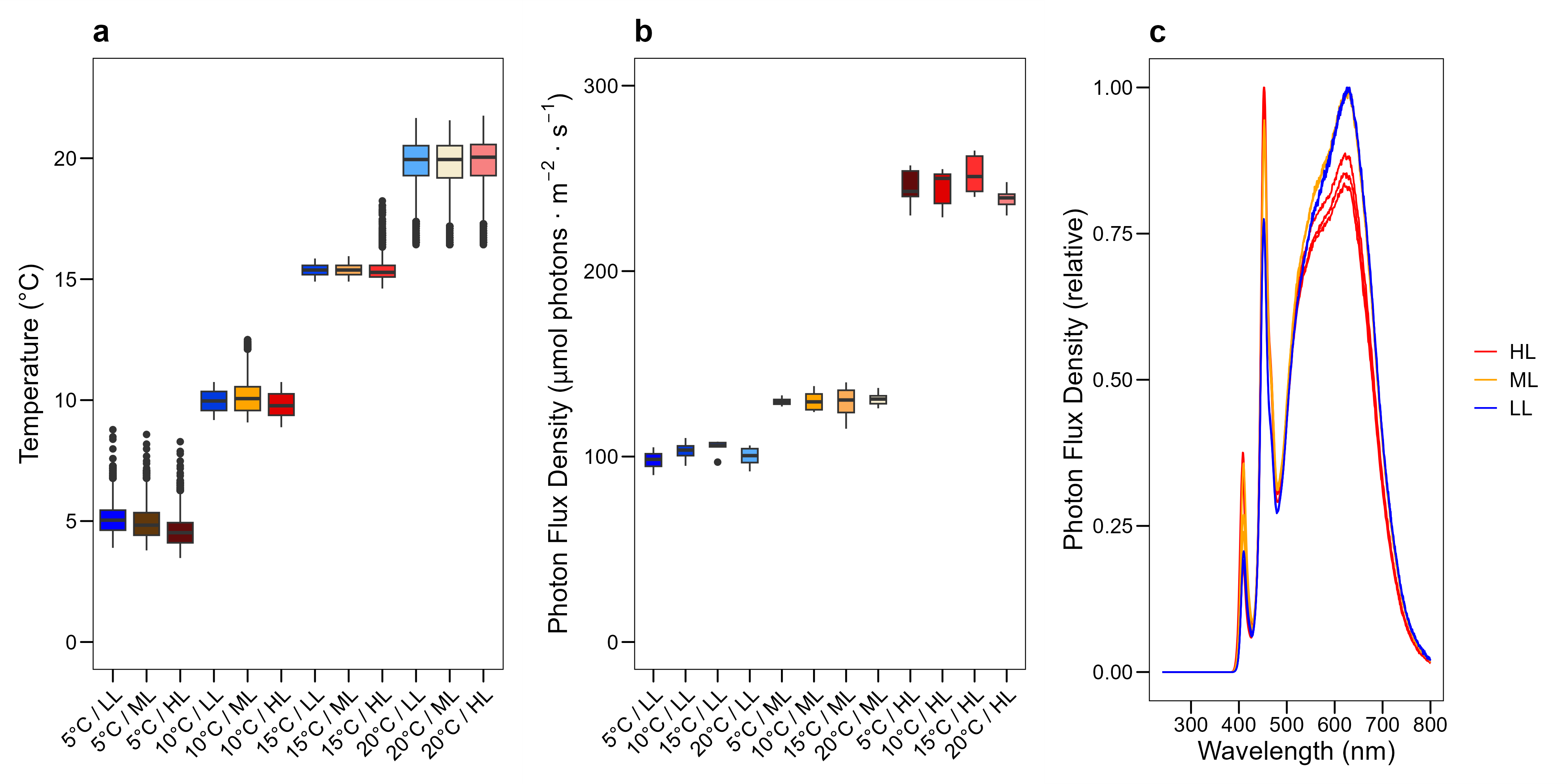

Supplement: Supplementary file 3 — Figure S3. Quantification of the cultivation system. (a) Water temperature (°C) of the cultivation vessels measured every 15 min during the experiment. (b) Photon flux density of PAR (μmol photons · m−2 · s−1) at the position of the culture vessel measured at cooling water level. (c) Spectrum of the light levels measured in the center and at the edge area of the cooling tubs. The box plots cover the 25th–75th percentiles, with black lines indicating the median. Whiskers extend to 1.5 times the interquartile range, with outliers shown as black dots. [file JPY-61-1863-s002.tif]

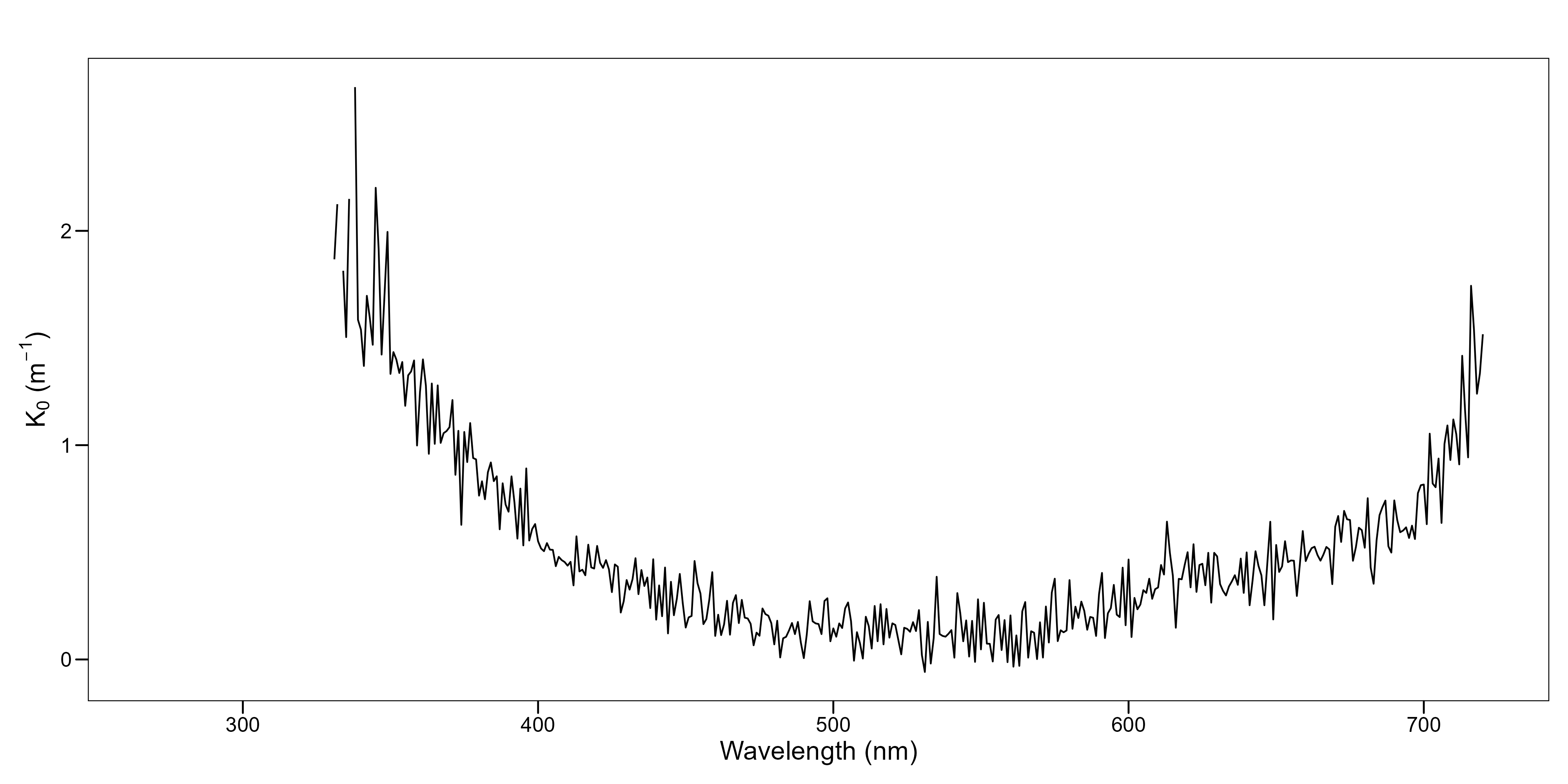

Supplement: Supplementary file 4 — Figure S4. Attenuation spectrum of Lake Torneträsk. [file JPY-61-1863-s001.tif]

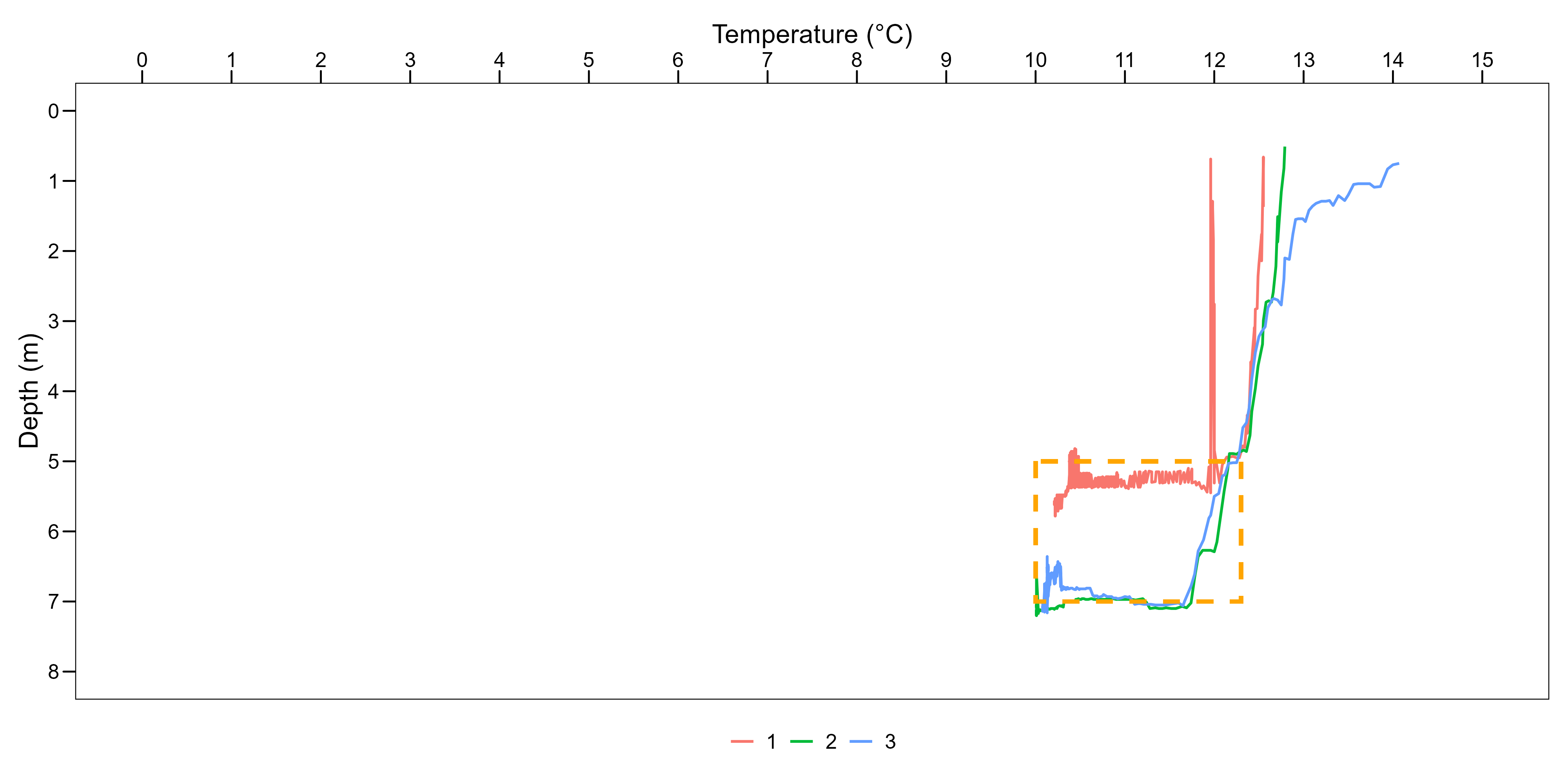

Supplement: Supplementary file 5 — Figure S5. Temperature and depth profiles recorded during the drone dives. S. canadensis was observed between ~5 and 7 m at three different sites, where water temperature ranged from 10°C to 12.3°C. The orange box indicates the area of occurrence. [file JPY-61-1863-s006.tif]

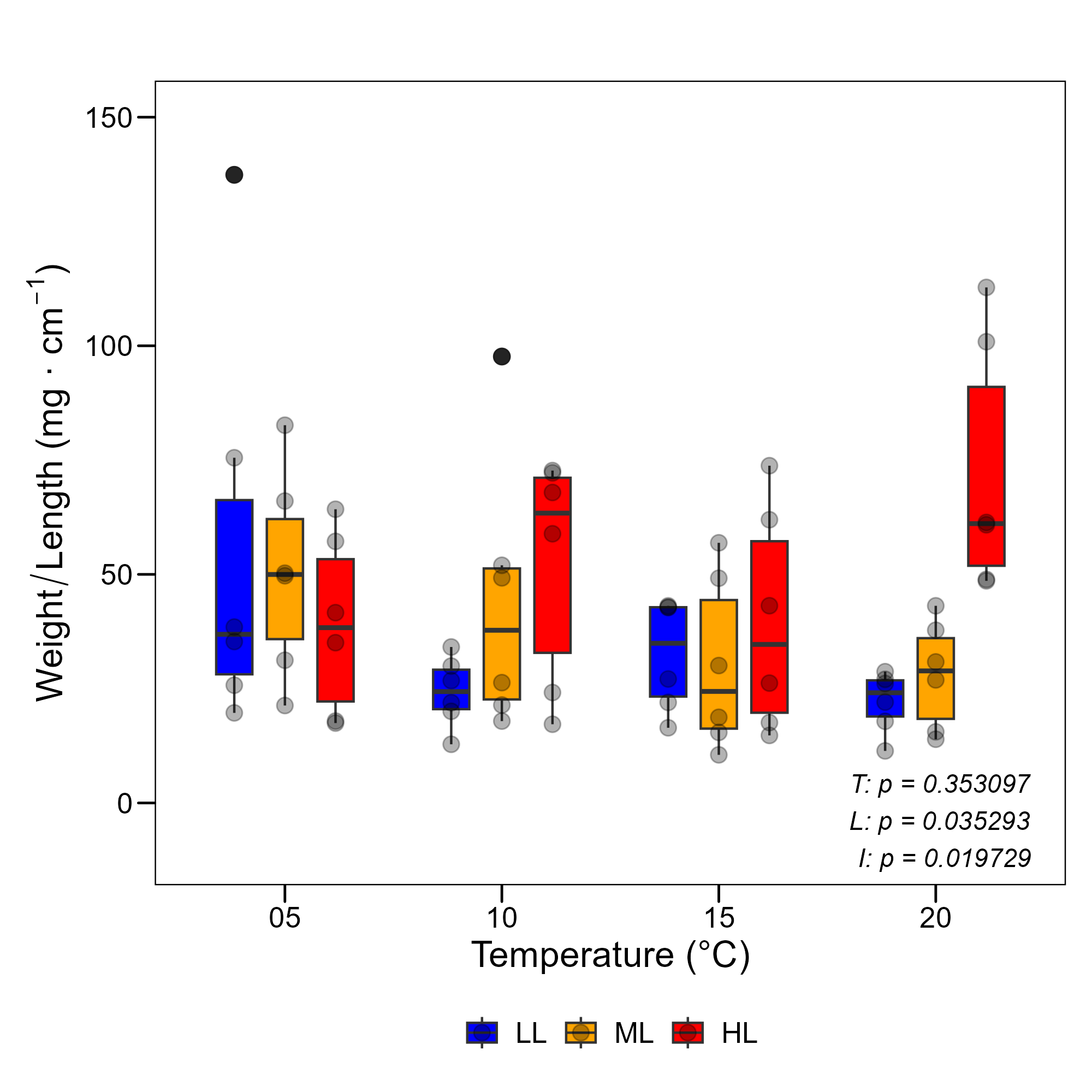

Supplement: Supplementary file 6 — Figure S6. Weight/length ratio (mg · cm−1) of Sphaerochara canadensis at the end of the cultivation experiment. The box plots cover the 25th–75th percentiles, with black lines indicating the median. Whiskers extend to 1.5 times the interquartile range, with outliers shown as black dots. Semi‐transparent black dots represent individual data points. Statistical results for the main effects of temperature (T), light (L), and interaction effect (I) are shown at the bottom right (permutation ANOVA, p < 0.05 considered significant). [file JPY-61-1863-s005.tiff]
