# Supplementary material for: Sphaerochara canadensis (Charophyceae): A circumpolar species with a high temperature optimum
Source: J Phycol. 2025 Dec 3;61(6):1863–73. doi: 10.1111/jpy.70111 (PMC12718439; doi:10.1111/jpy.70111)
Supplement: Supplementary file 7 — Table S1. eDNA sampling sites and results. [file JPY-61-1863-s003.docx]

| **combined**  **sample** | **locality** | **long** | **lat** | **temperature (°C)** | **result** |
| --- | --- | --- | --- | --- | --- |
| 1 | Lake Traunsee, Swimming area "Freisitz" | 13,80820 | 47,90811 | 10 | negative |
|  | Lake Traunsee, Swimming area "Umkehrplatz" | 13,81460 | 47,87297 | 10 |  |
|  | Lake Traunsee, Dive site "Zigeunerfeuchte" | 13,77887 | 47,87141 | 10 |  |
|  | Lake Traunsee, Dive site "Sonnstein” | 13,78683 | 47,83187 | 10 |  |
| 2 | Lake Attersee, Dive site "3 Tannen" | 13,50518 | 47,79012 | 11 | negative |
|  | Lake Attersee, Stockwinkl | 13,52369 | 47,83855 | 11 |  |
|  | Lake Attersee, Seewalchen | 13,59395 | 47,94932 | 11 |  |
|  | Lake Attersee, Weyregg, Dive site "Steinwand" | 13,56954 | 47,88297 | 11 |  |
| 3 | Lake Wolfgangsee, Fürbergbucht | 13,39134 | 47,76489 | 9 | negative |
|  | Lake Wolfgangsee, Swimming area "Franzosenschanze" | 13,39053 | 47,74189 | 9 |  |
| 4 | Lake Wolfgangsee, Bürglstein | 13,47621 | 47,72463 | 9 | negative |
| 5 | Ischler Ache just east of the outlet of Lake Wolfgangsee | 13,48893 | 47,72235 | 9 | negative |
